# Aluminum

**Published:** 1888-10

**Authors:** C. C. Carroll


ARTICLE II.

### ALUMINUM.

BY C. C. CARROLL, M. D.

Aluminum, one of the sixty odd elements, began to attract the attention of scientific men as early as 1807, when Sir Humphrey Davy first called attention to this peculiar metal, and sought to isolate it from its oxidee, alumina, by decomposing it with the electric current, but without success.

In 1824, Oerstadt thought he had isolated aluminum by the decomposition of Al.2 Cl.6, a double chloride of Al., by the use of potassium, and produced a metal having the lustre and general appearance of tin. Wohler, following in the footsteps of Oerstadt, in 1827, did isolate Al. by decomposing Al.2 Cl.6 by the use of potassium (K.), producing a fine gray powder with a metalic lustre.

No farther mention is made of any advance step in aluminum isolation and production until 1845, when Wohler produced some globules of Al. by reducing Al.2 Cl.6 to a vapor and passing it over potassium confined in platinum vessels with which it became alloyed, causing it to be melted at a very high heat; nevertheless, he was enabled from these globules to determine most of the properties peculiar to aluminum.

From the literature of Al., it would seem that to H. St. Claire Deville belongs the honor of having first isolated nearly pure Al., by sodium, in 1854, and of determining its true properties.

He was given governmental aid by Napoleon III., at the chemical works of Javel, where were produced many objects of Al., which were exhibited at the Paris exposition, in 1855, where this metal first attracted attention as having a probable future as an article of commercial use in the arts.

The first notice of Al. appeared in American prints, in 1856, in a published article in the *Mining Magazine*, vol 7, page 317, in which mention is made of Deville having produced 50 or 60 pounds of metallic Al. which he sold for $100.00 per pound.

About this time Mr Monier, of Camden, N. J., it was claimed, had succeeded in producing metallic sodium, by a peculiar process, cheaply, thus insuring a cheap production of Al. that would cause its general introduction into the mechanic arts.

Over thirty years have passed since Monier's blighted promise, during which, at frequent intervals, has the announcement been made that the lucky man had been found who had made the valuable discovery whereby a cheap method of producing Al. was to be had.

As already indicated, this subject began with this century to attract the attention of eminent scientific men, and during the past eighty years have chemists and metallurgists sought to wrest from this coy mistress the secret of her loves and hates, with comparatively little success, until hope deferred leads us to fear the century will expire ere the entire solution of the Al. cheap production problem is realized.

The investigation of scientists would seem often to travel in a circle, from which, once described, it is difficult to depart, as at the present time we find the most hopeful promise for cheap Al. reduced by electricity, as first suggested by Sir Humphrey Davy. Another set of experimenters are to-day following in the wake of Wohler's sodium process, and are seeking for a method of cheapening the price of metallic sodium, which is now worth $5.00 per pound, a price too high for cheap production of Al. by this means.

Among the recent experimenters in this direction is H. Y. Castner, of N. Y., who claimed, some two years since, to have invented a furnace, by means of which, he was able to produce sodium for 25 cents per pound, which price would at once bring Al. to a correspondingly cheap rate.

Aluminum is the most universally distributed throughout the earth's crust of all metals, constituting the very basis of mountain, hill and valley of the rock-ribbed earth upon which we tread. Notwithstanding its universality, it is never found metallic as gold, silver and tin; neither is even a trace of it found in animal or vegetable composition. It is found in combination with oxygen, silicon, fluorine, and other bases, forming a numerous class of compounds, mostly silicates, such as albite, anorthite, orthoclase K. mica, Na mica, Li mica, Mg mica, which, by the action of frost, water and general atmospheric influences, are decomposed, the alkali being carried away or replaced, leaving the residue in the form of clay. The clays are formed into soils, making the earth porous and susceptible of bringing forth the vegetable and fruit in its season for the sustenance of the animal, to be again given back as food throughout the ever ceaseless round of feeding of animal and, vegetable, each upon the other, without waste or loss, with marvelous economy, throughout the organic kingdom.

Many of the aluminum compounds are dull in color, without special interest to the common observer, such as mica, felspar, gneiss, porphy, eurite, trachyte, &c., while others possess a beautiful lustre and brilliancy and are classed among the precious stones, such as the ruby, sapphire, garnet, cyanite, topaz, turquoise, &c., including a long list of aluminum compounds, among which are bauxite and cryolite, which are chiefly used in the production of aluminum at the present time.—*The Archives of Dentistry.*

